# Editorial: Differentiated Thyroid Cancer - Risk Adapted Therapy, Genetic Profiling and Clinical Staging

**DOI:** 10.3389/fendo.2021.755323

**Published:** 2021-10-08

**Authors:** Christoph Reiners, Valentina M. Drozd

**Affiliations:** ^1^ Department of Nuclear Medicine, University Hospital Würzburg, Würzburg, Germany; ^2^ International Foundation “Arnica”, Minsk, Belarus

**Keywords:** differentiated thyroid cancer, risk adapted therapy, genetic profiling, staging of the primary, staging of lymph nodes

## Introduction

The incidence of differentiated thyroid cancer (DTC) has steadily increased since the 1980´s (1, 2). This highly significant rise is thought to be primarily due to the increasing use of thyroid ultrasound screening leading to overdiagnosis and overtreatment of early stage papillary thyroid microcancers (microPTC) (3, 4). In contrast with the steep increases observed for incidence, long-term DTC mortality, being generally low, declined or stabilized in many countries worldwide during over two or three decades. Guidelines today, recommend non-aggressive treatment for early stage microPTC (5) or even active surveillance only, based on positive experiences made with this concept in Japan (6).

However, not all cancer registries worldwide recorded a significant increase of only small, early-stage tumours but a rise of larger, later-stage tumours too (1, 2, 7), which is contrary to the idea of the dominant impact of screening (8). In addition, in North America, Australia and Asia, the downward trend of thyroid cancer mortality levels off or slightly increases since around 2000 (1, 2). So not all thyroid cancers can be considered as “indolent” or “harmless” (8) and the challenge is to make decisions for therapy based on proper differentiation between low, intermediate and high-risk thyroid cancers which may be difficult even in small DTC (9).

Fifteen papers in this Research Topic address important questions, which have to be answered if the approach of “personalized” or “precision” medicine, which today is generally accepted, shall be applied to DTC too.

Very relevant issues to be solved relate to risk-benefit considerations covering a broad spectrum from small, non-aggressive papillary thyroid cancers (PTC) in young patients, to aggressive, metastasizing cancers in elderly people.

### Early Diagnosis - Risk Adapted Therapy

Krajewska et al., Gliwice Poland contribute a comprehensive review on: “Early diagnosis of low-risk papillary thyroid cancer results rather in overtreatment than a better survival” with more than 100 references. The main message of this paper is that there is overdiagnosis in (very) early stages of PTC cases stage pT1 worldwide, which need not be treated aggressively - if at all. But – on the contrary – the increasing detection of more advanced cases of PTC demands adequate treatment. Benefits and side effects of surgical therapy, I-131 treatment and thyroid hormone supplementation should be carefully considered. Finally, the authors state that “numerous clinical trials are needed to change the clinical management of low and intermediate risk PTC”.

Concerning the other side of the spectrum of DTC’s aggressiveness, Qiu et al. from Shanghai, China, present a paper on “Long-term Outcomes and Prognoses of Elderly Patients (≥65-years-old) with Distant Metastases from Well-differentiated Thyroid Cancer during Radioiodine Therapy and Follow-up”. In a study of 193 DTC patients ≥ 65 years of age, distant metastases are prevalent in 31% of the cases, which were followed-up for up to 24 years. The 5 and 10 year disease specific survival is reported to be 77% and 49% respectively. Gross thyroidal extension and I-131 responsiveness could be identified to be significant, independent prognostic indicators, thus confirming the effectiveness of radioiodine therapy (RIT) in patients with advanced thyroid cancer.

### Genetic Risk Stratification

To decide properly which treatment should be recommended to a given patient, it is necessary to stage the tumour properly and to assign this clinical stage to defined risk categories. This today should be done including appropriate instruments of genetic profiling. Zhao et al., Hangzhou China present “Identification of A Prognostic 3-Gene Risk Prediction Model for Thyroid Cancer” using 506 DTC samples and 56 controls from the TCGA cancer genome database. Using receiver operator characteristics (ROC) to test predictability of 3-year survival, the area under the curve (AUC) corresponds remarkably to 0.85 for the 3 gene model with GHR, GPR125 and AtP2c2 versus AUC´s of 0.79-0.84 for single genes. GHR is a gene coding for transmembrane receptors of growth hormone, GPR125 promotes cell adhesion and AtP2c2 is a calcium transport coding gene.

Similarly, Xie et al. from Shenyang, China, used the same TCGA database for “Analysis of the prognostic value and potential molecular mechanisms of TREM-1 overexpression in papillary thyroid cancer *via* bioinformatics methods”. They focus on the triggering receptor expressed on myeloid cells-1 (TREM-1) described as biomarker in many cancers, but not up to now in thyroid cancer. Genes co-expressed with TREM-1 participate in immune related pathways. Xie et al. identified TREM-1 expression to be correlated with immune infiltration, tumour progression, and poor overall survival, with an AUC of 0.84. These interesting results obtained with bioinformatics methods have to be confirmed with much larger samples and experimental research.

### Staging the Primary, Uni Versus Bilateral PTC

PTCs are frequently diagnosed by chance if a non-suspicious nodule is surgically resected or lobectomy is performed for non-oncological reasons. In this case it is of utmost importance to know if the cancer disease is restricted to the lobe surgically resected or if possibly the contralateral lobe is affected too.

Feng et al., Changzhou, China, present a study on “Management of Clinically Solitary Papillary Thyroid Carcinoma Patients According to Risk-scoring Model for Contralateral Occult Carcinoma” in which 573 clinically solitary PTC patients undergo lobectomy, identifying 3.7% ipsilateral and 15,5% contralateral occult cancers with their approach. The 10-point risk scoring model for contralateral cancer includes the points “benign nodule (point-load 2)”, “tumour size > 1cm (load 1)”, “extrathyroidal extension (load 3)”, “central LN-metastases (load 2)”, “lateral LN-metastases (load 2)”, the ROC area under the curve amounting to 0.91 for a cut-off of 3.5 summarized points.

Zhang et al., Shenyang, China, contribute to the same Research Topic with a paper on “Risk Factors for Contralateral Occult Carcinoma in Patients with Unilateral Papillary Thyroid Carcinoma: A Retrospective Study and Meta-Analysis”. The meta-analysis in 4,347 patients includes 26% occult contralateral cancers. Significant risk factors are “tumour size > 1cm” with an odds ratio (OR) of 2.16, “central neck lymph node metastases” OR 2.80 and “multifocality ipsilateral” OR 5.62. Sex, age, ETE, capsular invasion, Hashimoto´s disease and lateral lymph node metastases are not described as risk factors for contralateral occult carcinoma.

### Staging the Lymph Nodes

Today’s guidelines for treatment of early-stage DTC (5, 10) strongly rely on lymph node staging. In this context it is important to use up-to-date diagnostic procedures like Colour-Doppler, or Contrast-Enhanced-Ultrasound. Eight papers of this section address multiparameter approaches to predict lymph node metastases (LNM) in different anatomic locations of the neck.

*“*Predictive factors of lymph node metastasis in patients with papillary microcarcinoma of the thyroid: a retrospective analysis on 293 cases” is the title of a paper by Medas et al. from Cagliari, Italy. An incidence of 13.7% of LNM (in any location) is described in their single hospital study in 293 PTC patients. Independent risk factors are: “age < 45”, “tumour size > 6 mm”, “tall cell variant”, “extrathyroidal extension (ETE)” and “angioinvasion”. The authors conclude that “size of the tumour being the most significant predictive factor” and that “smaller size of the tumour should be considered for active surveillance strategies”.

Two papers focus on preoperative prediction of central lymph node metastases. Chen et al. from Changsha, China, describe “Sonographic characteristics of papillary thyroid carcinoma with coexistent Hashimoto’s thyroiditis in the preoperative prediction of central lymph node metastasis”. They report a single hospital study of 177 patients with histologically verified PTC and Hashimoto Thyroiditis using colour-doppler (CDUS) and contrast enhanced ultrasound (CEUS). Risk factors for central lymph node metastases (CLNM) by univariate statistics are “age < 45” (P=0.03), “tumour size >10mm” (P<0.0001), “shape wider than tall” (P=0.0019), “CEUS hypoenhancement” (P=0.012), “CDUS peak intensity < 1” (P=0.11). Using multivariate logistic regression, the most significant risk factors for CLNM are “tumour size > 10mm” (OR 4.3, P< 0,0001) and “CEUS hypoenhancement” (OR 2.9, P =0,002).

Xue et al. from Changchun, China, include strap muscle infiltration (SMI) as risk factor, studying “Predictive Factors of Central-Compartment Lymph Node Metastasis for Clinical N0 Papillary Thyroid Carcinoma with Strap Muscle Invasion”. Their single hospital study comprises the considerable number of 9,866 PTC patients recruited between 2009 and 2017, among them 281 with SMI and 50.7% with CLNM. Risk factors for CLNM in patients with SMI turn out to be “male gender” with an OR of 6.22 (P<0,02) and “age < 40” OR=9.94 (P<0,001).

LNM of PTC anatomically close to the right recurrent laryngeal nerve (RLN) represent a special challenge for the surgeon. In their paper “Metastasis of cN0 Papillary Thyroid Carcinoma of the Isthmus to the Lymph Node Posterior to the Right Recurrent laryngeal Nerve”, Du et al. from Zhengzhou, China, investigate risk factors for such LNM in patients who presented clinically without central LNM. Their single hospital study comprises 357 patients who underwent bilateral central lymph node dissection. LNM posterior to the right RLN are described in 23 cases (6.7%) and only, when other LNM were prevalent (especially of the anterior right RLN with an OR of 6.9). Other significant independent factors were “tumour size > 5mm” (OR=2.8), “multifocality” (OR=2.2), and extrathyroidal extension (ETE) (OR=5.8). Zheng et al., Yantai, China focus on the “Clinical relevance and management of recurrent laryngeal nerve inlet zone lymph nodes metastasis in papillary thyroid cancer”, defining the zone of 10 mm around the inlet of the RLN as “inlet zone”. In their single hospital study of 947 patients with PTC, 150 received lymph node dissection, among them 47 (31%) present with such RLN inlet zone LNM. Additional ipsilateral LNM turn out to be highly predictive for RLN inlet zone LNM (OR 7.1). The authors conclude that “Once central or lateral LNM are confirmed preoperatively, RLN inlet zone dissection should be carefully performed to reduce the rate of structural recurrence in the central compartment”.

A more advanced approach for lymph node staging is to combine clinical and sonographic parameters in a model with a whole set of findings. This can be done using relatively simple nomograms or better applying recent advances of artificial intelligence and machine learning. The first two among three papers on nomograms for PTC consider all stages of primaries but focus on central LNM, whereas the last one focuses on microPTC considering all possible locations of LNM.

Li et al. from Tianjin, China, describe a “Diagnostic model incorporating clinicopathological characteristics of Delphian lymph node metastasis risk profiles in papillary thyroid cancer” The Delphian lymph node is located pretracheally and considered an effective indicator of aggressive disease and recurrence. In their single hospital study with 936 PTC patients, 177 presented with LNM to the prelaryngeal nodes (18.9%). Risk factors by multivariate statistics are “male gender” (OR 4.1, P <0.0001), “younger age (OR 1.0, P =0.0039), “larger tumour size” (OR 1.1, P <0.0001), “ETE” (OR 2.2, P=0.008), “lymphovascular invasion” (OR 4.4, P=0.007), “central LNM” (OR 4.5, P< 0.0001). The proposed nomogram to predict LNM to the Delphian node is considered to be a clinically sensitive predictor of further LN in the central compartment” (ROC AUC 0.75; verified in an independent cohort).

Feng et al., Changzhou, China, present “A Nomogram Based on Clinical and Ultrasound Characteristics to Predict Central Lymph Node Metastasis of Papillary Thyroid Carcinoma”. This has been developed in single hospital study with 886 PTC patients with LNM in 50% of the cases. It has to be emphasized that two different subsets of the cohort were used for training (n=617) and validation (n=269). Clinical and US variables are tested as potential predictors for central LNMs: “male sex” (OR 2.8, P<0.001), chronic lymphocytic thyroiditis (OR 1.9, P<0.001),”tumour size > 1cm” (OR 2.4, P<0.001), “tumour size > 2cm” (OR 4.0, P <0.001), “irregular margin” (OR 2.3, P <0,.001), “tumour location middle/lower quadrant” (OR 3.6, P< 0.001). ROC analysis results in AUCs for training of 0.81 and for validation of 0.80 respectively.

The third paper on nomograms by Sun et al., Wuxi, China, “Nomogram for preoperative estimation of cervical lymph node metastasis risk in papillary thyroid microcarcinoma” focuses on micro-PTC, but not on central LNM only, as the previous papers. The single hospital study comprises 552 patients, among them 61% with LNM. Seven variables of clinical and US features are identified as potential predictors including “male sex” (OR 2.0, P =0.004), “age < 45 years” (OR 4.6, P < 0.001), “US-reported central LN status” (OR 1.9, P =0.005), “multifocality of the tumour “ (OR 1.8, P =0.007), “tumour size ≥ 0.6cm” (OR 1.7, P =0.018), “ETE” (OR 3.8, P< 0.001) and “microcalcification by US” (OR 2.3, P < 0.001). In this study ROC analysis results in an AUC of 0.84.

According to a recent review, artificial intelligence and machine learning are very promising methods to improve the diagnostic sensitivity and specificity of ultrasound thyroid imaging (11). Up to now, the focus has been mainly on specification of malignancy in thyroid nodules. The following contribution of Wu et al. from Beijing, China, on “Machine learning algorithms for the prediction of central lymph node metastasis in patients with papillary thyroid cancer” takes another approach. The single hospital study in 1,103 PTC patients aims at predicting central LNM comparing seven different machine learning approaches and a set of 22 variables (including US parameters plus clinical data). For prediction of central LNM, a seven parameter machine learning gradient boosting decision tree, works best by including male sex, young age, low TPO-AB, US suspected LNM, microcalcifications, tumour size > 1.1 cm and TSH. The AUC is 0.73 in the training dataset, but no validation in a separate dataset has been performed up to now. Such approaches will gain rapidly increasing clinical relevance, provided that the data sets used are well defined, much larger and separate as well as independent sets are used for training and validation. Only cooperative networks of clinicians and computer scientists can meet these challenges.

## Author Contributions

Both authors contributed equally. All authors contributed to the article and approved the submitted version.

## Conflict of Interest

The authors declare that the research was conducted in the absence of any commercial or financial relationships that could be construed as a potential conflict of interest.

## Publisher’s Note

All claims expressed in this article are solely those of the authors and do not necessarily represent those of their affiliated organizations, or those of the publisher, the editors and the reviewers. Any product that may be evaluated in this article, or claim that may be made by its manufacturer, is not guaranteed or endorsed by the publisher.
